# Amplified RLR signaling activation through an interferon-stimulated gene-endoplasmic reticulum stress-mitochondrial calcium uniporter protein loop

**DOI:** 10.1038/srep20158

**Published:** 2016-02-19

**Authors:** Jinbo Cheng, Yajin Liao, Lujun Zhou, Shengyi Peng, Hong Chen, Zengqiang Yuan

**Affiliations:** 1 The State Key Laboratory of Brain and Cognitive Sciences, Institute of Biophysics, Chinese Academy of Sciences, Beijing 100101, China; 2 The College of Life Sciences, University of Chinese Academy of Sciences, Beijing 100049, China

## Abstract

Type I interferon (IFN-I) is critical for a host against viral and bacterial infections *via* induction of hundreds of interferon-stimulated genes (ISGs), but the mechanism underlying the regulation of IFN-I remains largely unknown. In this study, we first demonstrate that ISG expression is required for optimal IFN-β levels, an effect that is further enhanced by endoplasmic reticulum (ER) stress. Furthermore, we identify mitochondrial calcium uniporter protein (MCU) as a mitochondrial antiviral signaling protein (MAVS)-interacting protein that is important for ER stress induction and amplified MAVS signaling activation. In addition, by performing an ectopic expression assay to screen a library of 117 human ISGs for effects on IFN-β levels, we found that tumor necrosis factor receptor 1 (TNFR1) significantly increases IFN-β levels independent of ER stress. Altogether, our findings suggest that MCU and TNFR1 are involved in the regulation of RIG-I-like receptors (RLR) signaling.

The robust and rapid induction of IFN-I is important for a host in protection against viral and bacterial infections^1^. Upon infection with an RNA virus, virus-related molecules are recognized by retinoic acid inducible gene-I (RIG-I) and melanoma differentiation associated gene 5 (MDA-5), two receptors of the RIG-I-like receptor (RLR) pathway, leading to the production of IFN-I in most cell types. Upon activation, RIG-I or MDA5 binds to its adaptor mitochondrial antiviral signaling protein (MAVS, also referred to as IPS-1, VISA or Cardif) *via* CARD-CARD domain interaction. MAVS complexes then activate IFN-I by facilitating the nuclear translocation of IRF3^2^,^3^,^4^,^5^. The induction of IFN-I by the RLR pathway further regulates hundreds of IFN-stimulated genes (ISGs) by binding to interferon-α/β receptor 1 (IFNAR1) and activating downstream signaling^6^,^7^. However, despite decades of intensive studies, the precise mechanisms by which cellular IFN-I is robustly and rapidly induced have remained obscure.

The endoplasmic reticulum (ER) is a membrane-bound compartment that is important for many cellular functions, including protein processing and calcium homeostasis^8^,^9^,^10^. Interestingly, multiple lines of evidence show that virus infection always induces ER stress in mammalian cells^11^,^12^,^13^,^14^, and in turn, the ER stress-induced unfolded protein response (UPR) influences the expression of certain inflammatory cytokines^14^,^15^. Moreover, the ER is the main intracellular reservoir for calcium, and many ER physiological functions are calcium dependent^16^,^17^,^18^. However, it remains unclear whether ER stress is involved in the robust induction of IFN-I.

In this study, we found that ISG expression positively regulates IFN-β via ER stress. Furthermore, we employed a tandem affinity purification method and discovered a protein interacting with MAVS, mitochondrial calcium uniporter protein (MCU), thus linking ER stress with the MAVS-mediated immune response. In addition, by screening a library of 117 human ISG genes, we identified TNFR1 as a positive regulator of IFN-I expression.

## Results and Discussion

### ISG expression positively regulates IFN-β *via* ER stress

**Figure 1:**
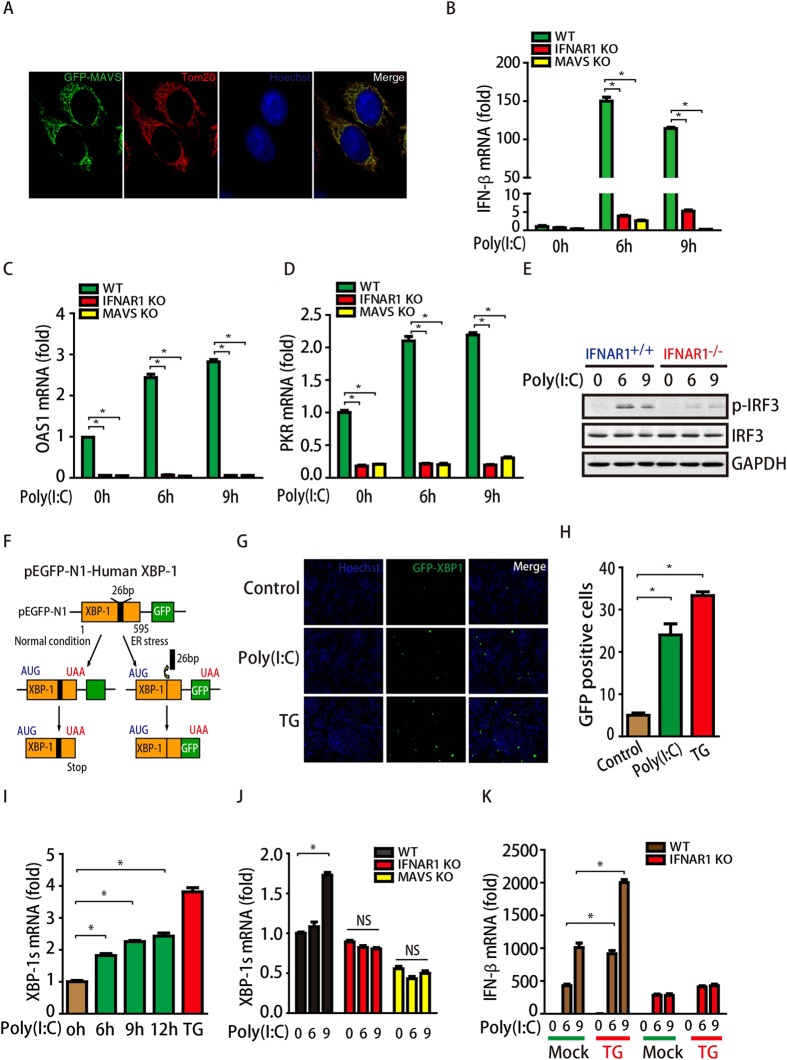
ISG expression positively regulates IFN-β via ER stress. (**A**) HeLa cells were transfected with GFP-MAVS and then immunofluorescence stained for Tom20 (Red). (**B**–**D**) WT, *MAVS*^*−/−*^, and *IFNAR1*^*−/−*^ MEF cells were treated with 1 μg/mL poly(I:C) for different times, as indicated, and IFN-β, OAS1 and PKR mRNA levels cells were detected by qPCR and normalized to GAPDH. (**E**) WT, and *IFNAR1*^*−/−*^ MEF cells were treated with 1 μg/mL poly(I:C) for different times, as indicated, and p-IRF3, IRF3 and GAPDH were detected by immunoblotting. ISG expression regulates IFN-β. (**F**) Schematic of the ER stress indicator pEGFP-N1-Human XBP1. (G, H) HeLa cells were transfected with pEGFP-N1-Human XBP1 and then treated with 1 μg/mL poly(I:C) for 24 h or TG (1 μM) for 6 h. GFP-positive cells were then detected and counted under a microscope. (I) HeLa cells were treated with 1 μg/mL poly(I:C) for 24 h or TG (1 μM) for 6 h, and XBP-1s mRNA was detected by qPCR. (J) WT, *MAVS*^*−/−*^, and *IFNAR1*^*−/−*^ MEF cells were treated with 1 μg/mL poly(I:C) for different times, and the IFN-β mRNA levels in these cells were detected by qPCR. (K) WT and *IFNAR1*^*−/−*^ MEF cells were treated with TG (1 μM) for 6 h prior to treatment with 1 μg/mL poly(I:C) for different times, as indicated; the XBP-1s mRNA levels in those cells were detected by qPCR.

Due to the critical role of IFN-I in the antiviral response and immune homeostasis^19^,^20^,^21^,^22^, it is worth clarifying the signaling factors involved in the rapid and robust induction of IFN-I in cells upon virus and bacterial infections. Previous studies have shown the existence of positive feedback pathways from ISG expression to the upstream cytokine IFN-β^23^,^24^,^25^,^26^. However, whether positive feedback pathways are also involved in RLR signaling has yet to be clarified. In the present study, we found that MAVS co-localized with Tom20 (a mitochondrial marker), suggesting that MAVS is expressed on mitochondria in HeLa cells (Fig. 1A), consistent with a previous report^4^. We also observed that the deletion of *MAVS* abolished poly(I:C)-induced IFN-β expression (Fig. 1B) and downstream ISG levels, including those of 2′,5′-oligoadenylate synthetase 1 (OAS1) and protein kinase R (PKR) (Fig. 1C,D). Interestingly, we found that knockout of *IFNAR1* significantly decreased IFN-β levels, suggesting that ISG expression positively regulates the upstream cytokine IFN-β. We also observed that deletion of *IFNAR1* dramatically reduced the levels of phosphorylated IRF3 upon poly(I:C) treatment. Thus, together with previous studies, we suggest that there is a feedback pathway from ISG expression to IFN-β in RLR signaling.

Accumulating evidence has shown that the virus infection-induced interferon response is always accompanied by ER stress in cells^11^,^12^,^14^. Thus, we addressed whether ISG expression regulates IFN-β through ER stress by constructing a GFP-tagged XBP-1 plasmid and transfecting it into HeLa cells. As shown in Fig. 1F, under normal conditions, XBP-1 is expressed without the GFP tag. However, when ER stress occurs, endoplasmic reticulum to nucleus signaling 1 (IRE1) removes a 26 bp intron from the Xbp1 transcript, leading to a translation frame-shift and fused expression of GFP. By using this system, we found that there were few GFP-positive cells under normal conditions. However, poly(I:C) or thapsigargin (TG, a specific ER stress inducer) treatment significantly increased the number of GFP-positive cells (Fig. 1G,H). Furthermore, we found that transcriptional XBP-1 splicing (XBP-1s) levels were also significantly increased in poly(I:C)- or TG-treated cells (Fig. 1I), suggesting that poly(I:C) treatment induces ER stress.

Next, we examined how poly(I:C) treatment induces ER stress. To address this, we first assessed whether MAVS or IFNAR1 is involved in this process. As shown in Fig. 1J, poly(I:C) treatment increased XBP-1s levels only in WT MEF cells but not in *MAVS*-knockout or *IFNAR1*-knockout cells, suggesting that poly(I:C) treatment-induced ER stress is indeed MAVS or IFNAR1 dependent. Given that the deletion of either *MAVS* or *IFNAR1* abolished ISG expression (Fig. 1C,D), these results indicate that poly(I:C)-induced ISG expression might be involved in the induction of ER stress. To further examine the role of ER stress in the activation of RLR signaling, MEF cells were treated with TG prior to poly(I:C) treatment. As shown in Fig. 1K, TG treatment significantly increased IFN-β levels in WT MEF cells, suggesting that ER stress positively regulates RLR signaling activation. However, this effect was largely inhibited in *IFNAR1*-knockout MEF cells, indicating that IFNAR1 signaling also regulates IFN-β expression through ER stress in an independent manner. Taken together, we found that ER stress is required for ISG expression-induced IFN-β production.

### MCU interacts with MAVS

**Figure 2:**
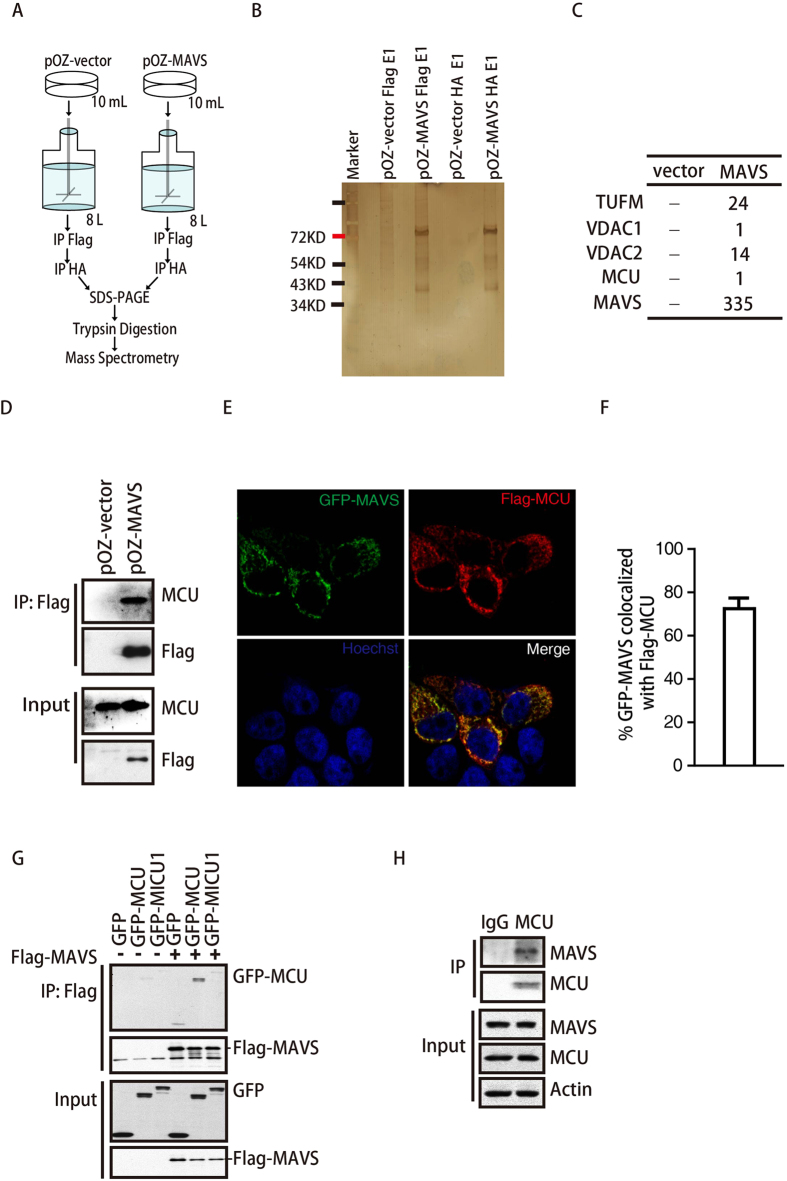
MCU interacts with MAVS. (**A**) Schematic and manufacturers of the series of affinity chromatography techniques used in this study. (**B**) Image of a silver-stained gel comparing different protein patterns in the control group and stable MAVS over-expression group. (**C**) Some MAVS-interacting proteins identified by mass spectrometry are listed. (**D**) Lysates of HeLa suspension cells stably over-expressing MAVS or control cells were immunoblotted using antibodies against Flag and MCU. (**E**,**F**) HeLa cells were transfected with GFP-MAVS and Flag-MCU, and 24 h later the cells were fixed and stained as indicated. The images were collected by the confocal laser scanning microscopy (Leica SP5 confocal microscope) using the X40 objective lens. The co-localization percentage of MAVS and MCU was analyzed by using Colocalizer pro software (20 cells were counted). (**G**) HEK293T cells were transfected with Flag-MAVS and GFP-MCU, GFP-MICU1 or the GFP vector, as indicated. Cell lysates were immunoprecipitated with anti-Flag M2 affinity gel and then immunoblotted with anti-GFP and anti-Flag antibodies. (**H**) Lysates from HeLa cells were immunoprecipitated with anti-MCU antibody and then immunoblotted with anti-MCU and anti-MAVS antibodies.

ER stress always induces mitochondrial dysfunction, such as calcium overload and the accumulation of reactive oxygen species (ROS)^27^,^28^,^29^. As MAVS complexes are mainly localized on mitochondria and mitochondrial dynamics affect RLR signaling activation^30^,^31^, we hypothesized that some proteins on mitochondria might link ER stress to RLR signaling activation. To identify such molecules that might interact with MAVS and participate in the MAVS-mediated immune response, we employed the tandem affinity purification method to identify MAVS-interacting proteins. We constructed an expression vector (containing both Flag and HA tags) encoding full-length MAVS and established a stably expressing suspension HeLa cell line. As shown in Fig. 2A, 8 L of suspended cells were lysed, and the lysates were sequentially immunoprecipitated using anti-Flag- and anti-HA-coated beads. Finally, the eluted proteins were separately fractionated by SDS-PAGE (Fig. 2B) followed by high-performance liquid chromatography and mass spectrometry. Many protein peptides were found only in the MAVS overexpression group, including mitochondrial Tu translation elongation factor (TUFM), voltage-dependent anion-selective channel protein 1 (VDAC1), voltage-dependent anion selective channel protein 2 (VDAC2) and MCU (Fig. 2C). Among these, TUFM, VDAC1 and VDAC2 have already been reported to interact with MAVS^32^,^33^. In contrast, MCU, a mitochondrial calcium uniporter protein, was only recently discovered^34^,^35^. We further confirmed the interaction of MAVS and MCU in cells stably expressing MAVS (Fig. 2D), and immunofluorescence experiments showed that MCU co-localizes with MAVS in HeLa cells (Fig. 2E,F). Next, we found that the interaction between MAVS and MCU appeared to be specific, given that MAVS did not interact with mitochondrial calcium uptake 1 (MICU1), a protein regulating MCU (Fig. 2G). Furthermore, we observed that endogenous MCU interacted with endogenous MAVS (Fig. 2H). Together, these results indicate a physical interaction between MCU and MAVS and suggest that MCU might regulate the MAVS-mediated immune response.

### MCU mediates ER stress-induced RLR signaling activation

**Figure 3:**
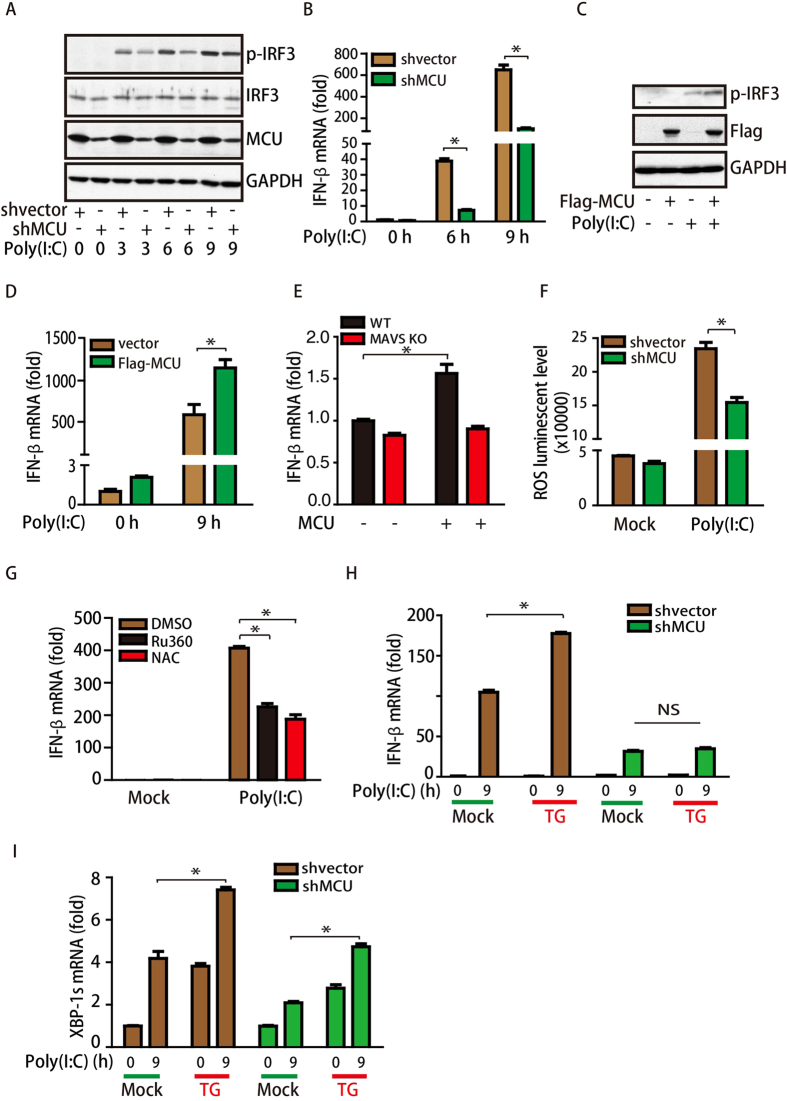
MCU mediates ER stress-induced RLR signaling activation. (**A**) Stable MCU-knockdown HeLa cells or control cells were treated with 1 μg/mL poly(I:C) for different times, and cell lysates were immunoblotted for p-IRF3, IRF-3, MCU and GAPDH. (**B**) Stable MCU-knockdown HeLa or control cells were treated with 1 μg/mL poly(I:C) for different times, as indicated, and IFN-β mRNA levels were detected by qPCR. (**C**) HeLa cells transfected with the Flag-MCU plasmid or the vector were treated with 1 μg/mL poly(I:C) for 6 h and then immunoblotted for p-IRF3 and GAPDH. (**D**) IFN-β mRNA levels in the cells treated with or without poly(I:C) were detected by qPCR. (**E**) WT and *MAVS*^*−/−*^MEF cells were transfected with Flag-MCU as indicated, and 24 h later the IFN-β mRNA levels in these cells were detected by qPCR. (**F**) Stable MCU-knockdown HeLa cells or control cells were treated with 1 μg/mL poly(I:C) for 24 h, and ROS levels in these cells were detected. (**G**) HeLa cells were treated with Ru360 and NAC prior to 1 μg/mL poly(I:C) treatment, and IFN-β mRNA levels in these cells were detected by qPCR. (**H**,**I**) Stable MCU-knockdown HeLa cells or control cells were treated with or without TG (1 μM) for 6 h prior to 1 μg/mL poly(I:C) treatment, and the mRNA levels of IFN-β and XBP-1s were detected by qPCR.

Because we identified MCU as a MAVS-interacting protein, we evaluated whether MCU is involved in MAVS-mediated immune activation. In control cells, poly(I:C) treatment induced a significant increase in the levels of phosphorylated IRF3, but this effect was largely inhibited in MCU-knockdown cells (Fig. 3A). A similar effect of MCU was observed with regard to IFN-β levels (Fig. 3B and Sup Fig. 1A,B). To further confirm this finding, we transfected Flag-tagged MCU into HeLa cells and examined the effect on the immune response. As shown in Fig. 3C,D, ectopic expression of MCU resulted in large increases in the levels of p-IRF3 and IFN-β. Consistently, similar observations were found for SeV infection-induced IFN-β levels and XBP-1s levels (Sup Fig. 2A–D). Furthermore, we found that the effect of MCU for IFN-β levels was MAVS-dependent, as knockout of *MAVS* disrupted this increase (Fig. 3E). Therefore, these results suggest that MCU positively regulates IFN-β levels in a MAVS-dependent manner.

Next, we asked how MCU regulates the MAVS-mediated immune response. As shown in Supplemental Fig. 3A, we found that MCU knockdown did not alter cell cycle parameters. However, knockdown of MCU did impair mitochondrial calcium uptake ability^36^ and decrease reactive oxygen species (ROS) levels induced by poly(I:C) treatment (Fig. 3F), which is reported to be essential for RLR signaling activation^37^. As MCU is a mitochondrial calcium uniporter protein, we then tested whether its calcium uniporter capacity was necessary for this process. We treated cells with Ru360, a specific mitochondrial calcium uptake inhibitor, to block calcium uniporter activity. Our results showed that Ru360 treatment significantly decreased poly(I:C)-induced IFN-β levels (Fig. 3G), suggesting that the regulation of MCU for the MAVS-mediated immune response is dependent on its capacity as a calcium uniporter. In addition, N-acetyl-L-cysteine (NAC, an inhibitor of ROS production) also reduced poly(I:C)-induced IFN-β levels (Fig. 3G). Lastly, we tested whether MCU is involved in the ER stress-induced upregulation of RLR signaling activation. As shown in Fig. 3H,I, MCU knockdown abolished TG treatment-induced IFN-β expression, suggesting that MCU contributes to the ER stress-induced amplification of RLR signaling activation. Together, these results indicate that ISG expression-ER stress-MCU constitute a positive feedback pathway that contributes to amplified IFN-β expression.

### TNFR1 is one ISG that positively regulates RLR signaling independent of ER stress

**Figure 4:**
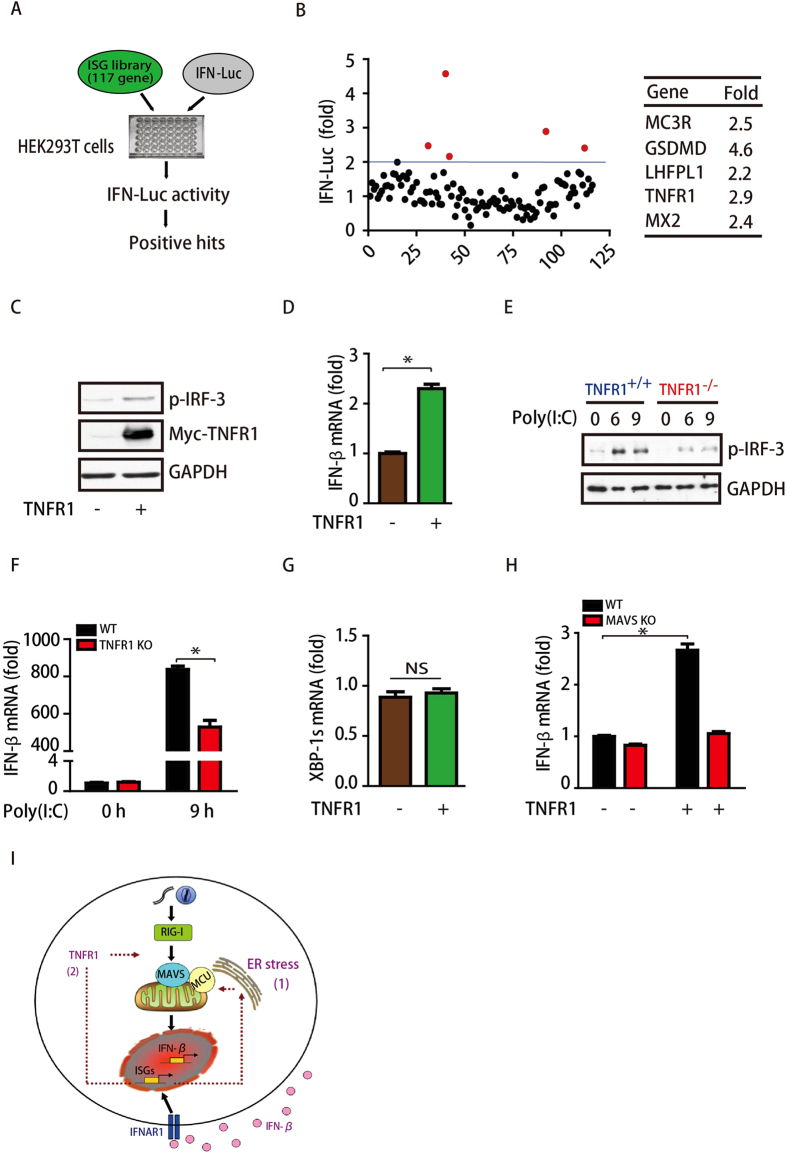
TNFR1 positively regulates RLR signaling. (**A**) Model of library screening for ISG genes with effects on IFN-β levels. (**B**) Each ISG gene was transfected into HeLa cells together with an IFN-β luciferase reporter, and five positive genes were found to increase the activity of the IFN-β promoter (>2-fold). (**C**,**D**) HeLa cells were transfected with Myc-TNFR1 and then immunoblotted for p-IRF3, RIG-I, Myc and GAPDH. The mRNA level of IFN-β in those cells was detected by qPCR. (**E**) WT and *TNFR1*^*−/−*^ MEF cells were treated with 1 μg/mL poly(I:C) for different times, as indicated, and then immunoblotted for p-IRF3, IRF-3, RIG-I and GAPDH. (**F**) WT and *TNFR1*^*−/−*^ MEF cells were treated with 1 μg/mL poly(I:C) for 9 h, and IFN-β mRNA levels in those cells were detected by qPCR and normalized to GAPDH. (**G**) HeLa cells were transfected with Myc-TNFR1, and the mRNA level of XBP-1s in these cells was detected by qPCR. (**H**) WT and *MAVS*^*−/−*^MEF cells were transfected with Myc-TNFR1 as indicated, and 24 h later the IFN-β mRNA levels in these cells were detected by qPCR. (**I**) Schematic model of positive feedback loops regulating RLR signaling activation.

To further define specific ISGs in the regulation of amplified IFN-β expression, we performed an ectopic expression assay to screen a library of 117 human ISGs for impacts on IFN-β levels. As shown in Fig. 4A, we transfected each ISG gene into HEK293T cells together with a luciferase reporter containing the proximal promoter sequence of IFN-β. Our results showed that five ISGs (MC3R, GSDMD, LHFPL1, TNFR1, and MX2) significantly increased IFN-β levels (>2-fold) (Fig. 4B). Subsequent functional studies revealed that the ectopic expression of TNFR1 significantly increases the levels of p-IRF3 and IFN-β (Fig. 4C,D). To confirm this, we isolated *TNFR1*-knockout MEF cells and observed that the knockout of this gene significantly decreased the poly(I:C)-induced levels of IRF3 phosphorylation and IFN-β mRNA (Fig. 4E,F). Together, our results indicate that TNFR1, as an ISG, positively regulates RLR signaling activation. We then assessed whether TNFR1 could activate RLR signaling through the induction of ER stress. Unfortunately, the ectopic expression of TNFR1 failed to induce ER stress in cells (Fig. 4G), indicating that TNFR1 positively regulates RLR signaling independent of ER stress. Interestingly, we found that the effect of TNFR1 for RLR signaling was also MAVS-dependent. As shown in Fig. 4H, knockout of *MAVS* significantly blocked overexpression of TNFR1-induced the increase of IFN-β levels. We also observed that RIG-I and MDA5, the key components of RLR signaling activation, were dramatically decreased in *IFNAR1* knockout MEF cells in a transcriptional-dependent manner (Sup Fig. 4A–C). Our findings support that ISG expression-induced ER stress might be mediated by the converged expression of multiple proteins, warranting further investigation.

In summary, we found that ISGs were required for IFN-β expression in response to infection, and the effect is further enhanced by ER stress. Furthermore, we identified MCU, the MAVS interacting protein, mediated the ER stress-induced RLR signaling activation. In addition, TNFR1, an ISG gene, also positively regulates the induction of IFN-β. Taken together, our findings demonstrate that MCU and TNFR1 were involved in the regulation of RLR signaling (Fig. 4I). The present work implicates potential targets in the treatment of IFN-I dysfunction-induced diseases.

## Methods

### Animals

Mice were maintained under conditions of a 12-h light/dark cycle at 23 °C with food and water ad libitum in the Animal Care Facility at the Institute of Biophysics (Beijing, China). All animal experiments were performed in accordance with the relevant guidelines and regulations that were approved by the Committee on Animal Care and Use of Institute of Biophysics, Chinese Academy of Sciences. China. *MAVS*^*−/−*^ mice^4^ and *IFNAR1*^*−/−*^ mice^38^ were kindly provided by Dr. Baidong Hou (Institute of Biophysics, Chinese Academy of Science, China). *TNFR1*^*−/−*^ mice were provided by Dr. Zhihai Qin (Institute of Biophysics, Chinese Academy of Science, China).

### Cell Culture

MEF cells were isolated from mouse embryonic tissue at E14.5 and cultured in DMEM supplied with 10% fetal bovine serum. HeLa cells and HEK293T cells were maintained in DMEM supplied with 10% fetal bovine serum at 37 °C in a humidified atmosphere with 5% CO_2._

### Reagents

Poly(I:C) was purchased from InvivoGen. The information of antibodies used in this study is as follows: Rabbit polyclonal anti-p-IRF3 (Ser396, Cell Signaling, 1:1000), anti-IRF3 (FL-425, Santa Cruz, 1:1000), anti-GAPDH (CW0100A, CWBiotech, 1:1000), anti-human MAVS (ab25084, Abcam, 1:1000), anti-RIG-I (ABclonal Technology, 1:500), anti-MDA-5 (ABclonal Technology, 1:500)

### Immunoprecipitation and Immunoblot Analysis

Briefly, cells were collected and lysed with IP buffer containing 0.5% NP-40, 150 mM NaCl, 50 mM Tris-HCl, PH 8, 50 mM NaF, and 2 mM EDTA, plus a protease inhibitor mixture (Roche Applied Science). Equivalent amounts of cellular extract were incubated overnight with antibody-coated Protein G Sepharose (GE Healthcare Life, 1 μg antibody and 25 μl Protein G Sepharose for each sample). The immunoprecipitates were washed four times in lysis buffer and eluted by boiling in Laemmli sample buffer (Bio-Rad). The samples were fractionated by SDS-PAGE and transferred to nitrocellulose. Immunoblots were probed with the indicated primary antibodies and visualized using ECL (Thermo).

### Immunofluorescence

HeLa cells were washed three times with PBS and then blocked with 5% goat serum in PBS containing 0.2% Triton X-100. The cells were then incubated with the primary antibody overnight at 4 °C. After washing three times with PBS, Alexa Fluor 546-conjugated secondary antibody (Invitrogen) was added and incubated for 1 h at room temperature. Finally, nuclear morphology was visualized using Hoechst 33258 (Sigma).

### siRNA- or shRNA-Mediated Gene Silencing

For siRNA transfection, HeLa cells were plated into 12-well plates and then transfected with 20 nM siRNA using Lipofectamine 2000 (Invitrogen) according to the manufacturer’s instructions (siRNA sequences are as follows: MCU#1 Sense: 5′-GCCAGAGACAGACAAUACUtt-3′; Antisense: 5′-CGGUCUCUGUCUGUUAUGAtt-3′; MCU#2 Sense: 5′-GGGAAUUGACAGAGUUGCUtt-3′; Antisense: 5′-CCCUUAACUGUCUCAACGAtt-3′). For selecting the stable MCU-knockdown cell line, the pLKO.1 vector containing shRNA was co-transfected with VSV-G and pCMV-dR8.12 into HEK293T cells, and virus was collected at 36 hr and 48 hr after transfection. Stable-knockdown cell lines were established by infecting the corresponding lentivirus, before selection in complete medium containing 2.0 μg/mL puromycin, as described previously^36^.

### Quantitative RT-PCR

Total RNA was extracted from cells using the Trizol reagent (Invitrogen). cDNA synthesis was performed using a one-step first-strain cDNA synthesis kit (Transgen). Quantitative real-time PCR was performed with primers for IFN-β (Human IFN-β: Forward: 5′-ATGACCAACAAGTGTCTCCTCC-3′; Reverse: 5′-GGAATCCAAGCAAGTTGTAGCTC-3′; Mouse IFN-β: Forward: 5′-GCACTGGGTGGAATGAGACTATTG-3′; Reverse: 5′-TTCTGAGGCATCAACTGACAGGTC-3′), Mouse OAS1 (Forward: 5′-GCTGTGGTACCCATGTTTTATGAA-3′; Reverse: 5′-AACCACCGTCGGCACATC-3′), Mouse PKR (Forward: 5′-CCGAAAACTGCCGGAACA-3′; Reverse: 5′-CTGACTGGGAAACACCATTACTTG-3′), XBP-1s (Human XBP-1s: Forward: 5′-TGCTGAGTCCGCAGCAGGTG-3′; Reverse: 5′-GCTGGCAGGCTCTGGGGAAG-3′; Mouse XBP-1s: Forward: 5′CTGAGTCCGCAGCAGGT-3′; Reverse: 5′-TGTCAGAGTCCATGGGAAGA-3′), Mouse RIG-I (Forward: 5′-AAGAGCCAGAGTGTCAGAATCT-3′; Reverse: 5′-AGCTCCAGTTGGTAATTTCTTGG-3′), Mouse MDA-5 (Forward: 5′-AGATCAACACCTGTGGTAACACC-3′; Reverse: 5′-CTCTAGGGCCTCCACGAACA-3′) and GAPDH (Human GAPDH Forward: 5′-GGAGCGAGATCCCTCCAAAAT-3′; Reverse: 5′-GGCTGTTGTCATACTTCTCATGG3′; Mouse GAPDH Forward: 5′-AGGTCGGTGTGAACGGATTTG-3′; Reverse: 5′-GGGGTCGTTGATGGCAACA-3′).

The RT-PCR reactions were performed using 2x SYBR Green PCR master mix (Transgen) and an Agilent Mx3005P RT-PCR system. The mRNA levels were normalized to GAPDH expression levels.

### ROS Detection

Cellular ROS were measured using a commercial kit (ROS-Glo^TM^ H_2_O_2_ Assay, Promega) according to the manufacturer’s instructions.

### Luciferase Assay

Luciferase activity was measured with the Dual-Luciferase reporter assay system according to the manufacturer’s protocol (Promega).

### Statistical Analysis

All values are expressed as the mean ± SEM. The statistical analysis was performed with the t-test for two groups or one-way ANOVA for multiple groups (Graphpad Software). A p value <0.05 was considered to be significant.

## Additional Information

**How to cite this article**: Cheng, J. *et al.* Amplified RLR signaling activation through an interferon-stimulated gene-endoplasmic reticulum stress-mitochondrial calcium uniporter protein loop. *Sci. Rep.* **6**, 20158; doi: 10.1038/srep20158 (2016).

## Supplementary Material

Supplementary Information
