# Supplementary material for: Amplified RLR signaling activation through an interferon-stimulated gene-endoplasmic reticulum stress-mitochondrial calcium uniporter protein loop
Source: Sci Rep. 2016 Feb 19;6:20158. doi: 10.1038/srep20158 (PMC4759556; doi:10.1038/srep20158)
Supplement: Supplementary Information [file srep20158-s1.pdf]

Supplemental information

Title: Amplified RLR signaling activation through an interferon-stimulated gene-endoplasmic reticulum stress-mitochondrial calcium uniporter protein loop

Jinbo Cheng[1,3], Yajin Liao[1,2,3], Lujun Zhou[1,2,], Shengyi Peng[1], Hong Chen[1], Zengqiang Yuan[1,*]

[1]The State Key Laboratory of Brain and Cognitive Sciences, Institute of Biophysics, Chinese Academy of Sciences, Beijing 100101, China

[2]The College of Life Sciences, University of Chinese Academy of Sciences, Beijing 100049, China

[3]Co-first author

*Corresponding author: Zengqiang Yuan, PhD. State Key Laboratory of Brain and Cognitive Sciences, Institute of Biophysics, Chinese Academy of Sciences, Beijing 100101, China. Email: zqyuan@ibp.ac.cn;

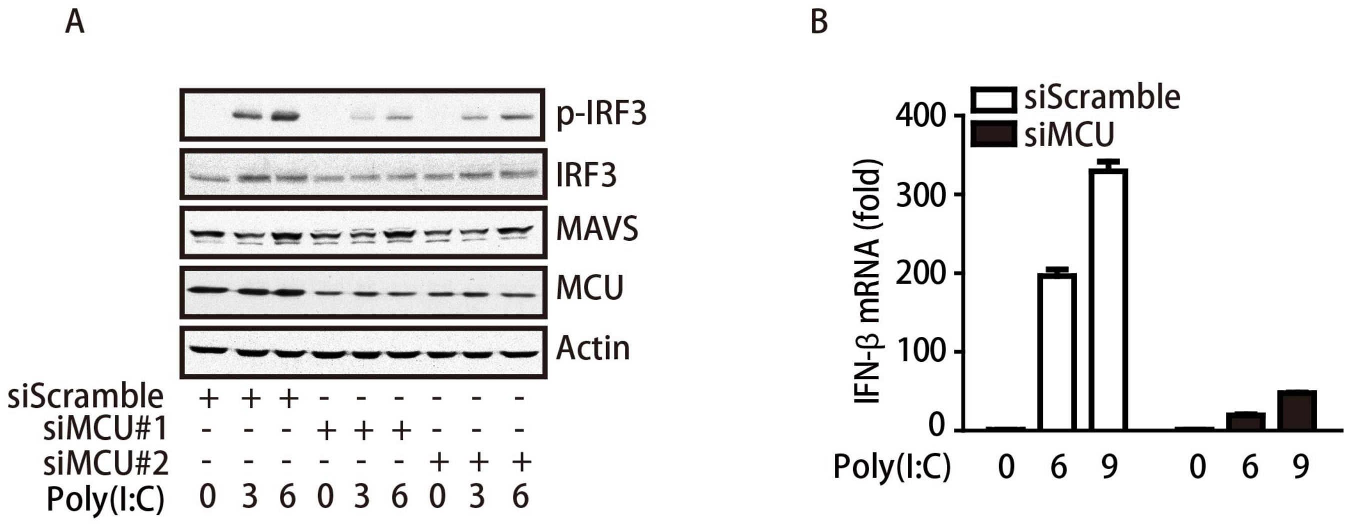
A
p-IRF3
IRF3
MAVS
MCU
Actin
siScramble + + + - - - - - -
siMCU#1 - - - + + + - - -
siMCU#2 - - - - - - + + +
Poly(I:C) 0 3 6 0 3 6 0 3 6
B
siScramble
siMCU
IFN-β mRNA (fold)
400
300
200
100
0
Poly(I:C) 0 6 9 0 6 9

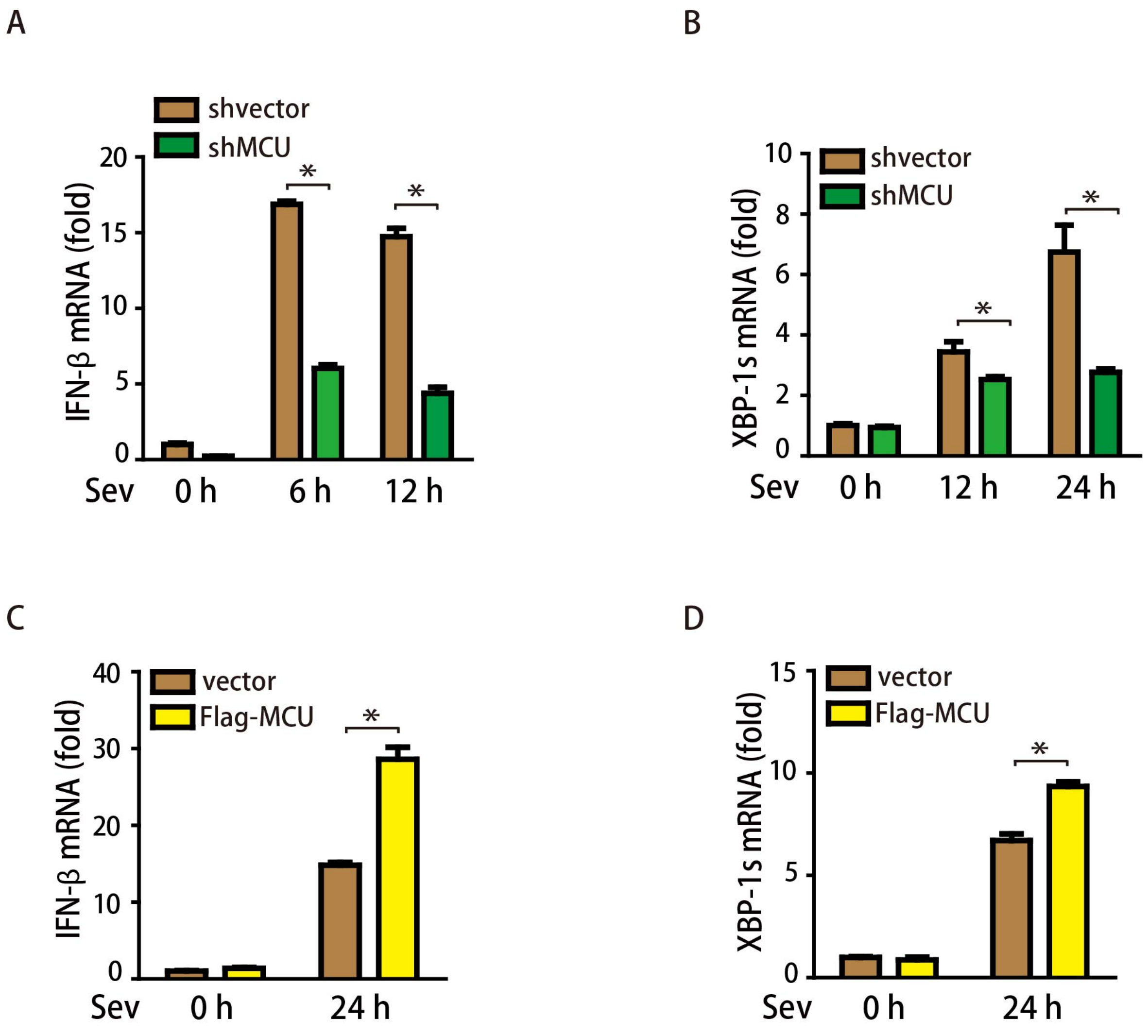
A
shvector
shMCU
IFN-β mRNA (fold)
20
15
10
5
0
*
*
Sev
0 h
6 h
12 h
B
shvector
shMCU
XBP-1s mRNA (fold)
10
8
6
4
2
0
*
*
Sev
0 h
12 h
24 h
C
vector
Flag-MCU
IFN-β mRNA (fold)
40
30
20
10
0
*
Sev
0 h
24 h
D
vector
Flag-MCU
XBP-1s mRNA (fold)
15
10
5
0
*
Sev
0 h
24 h

A

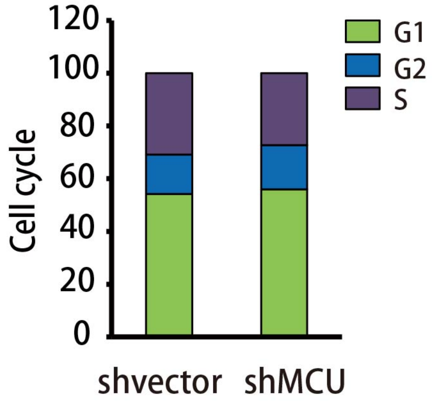

A

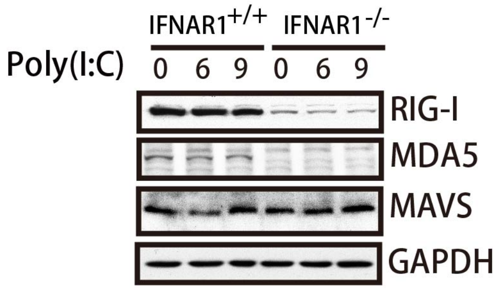
IFNAR1+/+
IFNAR1-/-
Poly(I:C)
0 6 9 0 6 9
RIG-I
MDA5
MAVS
GAPDH

B

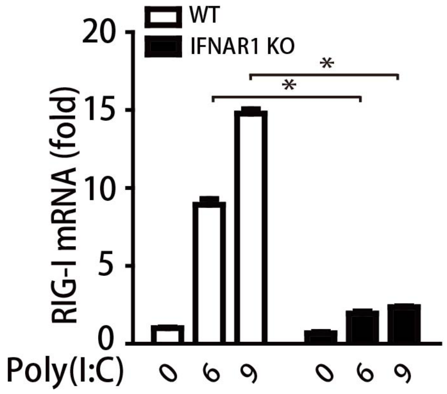
WT
IFNAR1 KO
RIG-I mRNA (fold)
20
15
10
5
0
*
*
Poly(I:C)
0 6 9 0 6 9

C

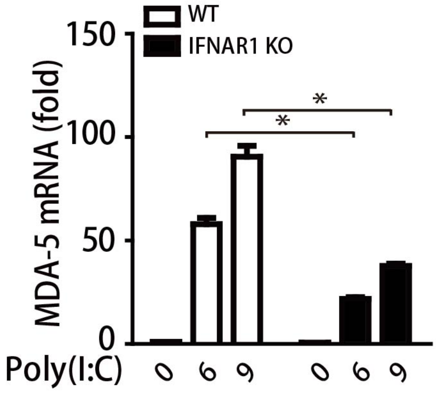
WT
IFNAR1 KO
MDA-5 mRNA (fold)
150
100
50
0
*
*
Poly(I:C)
0 6 9 0 6 9

Figure legends

Figure S1. Transient knockdown of MCU decreased poly(I:C)-induced RLR signaling activation. (A) HeLa cells were transfected with MCU targeting siRNAs (siRNA#1 and siRNA#2) and then treated with 1 μg/mL poly(I:C) for different time as indicated. Cell lysates from these cells were immunoblotted with p-IRF3, IRF-3, MAVS, MCU and Actin. (B) HeLa cells were transfected with MCU targeting siRNA#1 and then treated with 1 μg/mL poly(I:C) for different time as indicated. IFN-β mRNA levels from these cells were detected by qPCR.

Figure S2. MCU impairs SeV infection-induced RLR signaling activation and ER stress. (A, B) Stable MCU knockdown HeLa cells or control cells were treated with SeV for different time. IFN-β mRNA levels and XBP-1s levels from these cells were detected by qPCR. (C, D) HeLa cells transfected with Flag-MCU or vector were treated with SeV for different time, and then IFN-β mRNA levels and XBP-1s levels from these cells were detected by qPCR.

Figure S3. MCU knockdown did not impair cell cycle parameters. (A) Stable MCU knockdown HeLa cells or control cells were analyzed cell cycle by flow cytometry.

Figure S4. IFNAR1 signaling impairs the expressions of RIG-I and MDA-5. (A) WT and *IFNAR1*$^{-/-}$ MEF cells were treated with 1 μg/mL poly(I:C) for different time as indicated, and then immunoblotted with RIG-I, MDA-5, MAVS and GAPDH. (B-C) mRNA levels of RIG-I and MDA-5 from these cells were detected by qPCR.
